# Effects of temperature and salinity on metabolic rate of the Asiatic clam *Corbicula fluminea* (Müller, 1774)

**DOI:** 10.1186/2193-1801-3-455

**Published:** 2014-08-22

**Authors:** Bai-cai Xiao, Er-chao Li, Zhen-yu Du, Run-lan Jiang, Li-qiao Chen, Na Yu

**Affiliations:** School of Life Science, East China Normal University, Shanghai, 200240 China

**Keywords:** Temperature, Salinity, Body size, *Corbicula fluminea*, *Q*_10_ coefficient, O: N ratio

## Abstract

The effects of temperature and salinity on the metabolism of the Asiatic clam *Corbicula fluminea* (mollusca, Lamellibranchia) were studied experimentally. Firstly, three indexes of basal metabolism (oxygen consumption rate, OCR; ammonia excretion rate, AER; and CO_2_ emission rate, CER), patterns of diurnal rhythm and O: N ratios were measured for three size ranges (large: h = 25.54 ± 1.96 mm, medium: h = 22.07 ± 1.33 mm and small: h = 17.70 ± 1.43 mm) at two salinities (0.3‰ and 1.8‰). The results showed that: (1) three indexes decreased with increasing body size. (2) no significant difference was found between two salinities for the O: N ratios of the small and large size, but a significant difference was found for the medium-sized one; (3) however, there were similar and distinct diurnal rhythms of metabolic rate at two salinities over a 24 hour period in three size *C. fluminea*.

OCR, AER, CER, O: N ratios and Q10 (temperature coefficient) of small-sized *C. fluminea* were measured across five water temperatures (4, 11, 18, 25 and 32°C) and two salinities (0.3‰ and 1.8‰) in the following experiments. Our results of the small *C. fluminea* were as follows: there was no significant difference in the O: N ratios among the five temperatures and two salinity treatments; and no significant difference of three indexes between both salinity levels were observed at same temperature controlled; and three indexes increased significantly with increasing temperature from 4°C to 25°C, while no significant difference was observed in the 25-32°C range; and the highest Q_10_ coefficients (Q_10_ = 1.825 at salinity of 0.3‰ and Q_10_ = 1.683 at salinity of 1.8‰) were observed at the 18-25°C temperature increase, and the low values were found in the 4-11°C, 11-18°C and 25-32°C interval. It indicates that there is not a synergetic effect of our temperature and salinity on the metabolic rate of small *C. fluminea*, and a temperature of 18-25°C may represent an optimum adequate metabolic temperature range. For the purposes of ecological monitoring and restoration, small individuals of *C. fluminea* planted are more likely to survive than larger ones.

## Introduction

Natural estuarine processes are impacted by factors such as global temperature increase, sea level rise, saltwater intrusions and pollution, so that the ability of estuarine organisms to adapt to these changes can be sharply weakened. Saltwater intrusions are a basic characteristic of estuarine environments. With the rapid industrialization and urbanization of coastal areas, many estuaries are exposed to serious pollution, such as heavy metals, oils, persistent organic pollutants (POPs) and nutrients (Nelson et al. 1977; Sousa et al. 2008). The deterioration of water quality associated with these pollutants causes the destruction of estuarine habitats, leading to a reduction in biodiversity and the decline of fisheries resources. The majority of aquatic species do not have the ability to adapt to these environmental disturbances. To further our knowledge of these dynamic environments, it is necessary to select a type species that responds well to the variable processes and that can be used to determine levels of organic pollutants from both water and sediment.

Estuaries are constantly changing environments, and temperature and salinity are two of the most critical natural physical factors that affect aquatic animals (Paul 1980). Temperature can influence ingestion rates, feeding, respiration, metabolic activity, growth, reproduction and gametogenesis in poikilothermic organisms (Widdows and Bayne 1971; Navarro et al. 2000; González et al. 2002; Saucedo et al. 2004; Christophersen and Strand 2003). Temperature has also been observed to influence the filtration rate and assimilation efficiency of bivalves (Widdows and Bayne 1971). Similarly, salinity is a limiting factor in the distribution of aquatic organisms, and it can affect the physiological processes of estuarine organisms, such as survival, hemolymph osmolarity, tissue water content and have other sublethal effects (De Lisle and Roberts 1988; Matsuda et al. 2008; Taware et al. 2012; McFarland et al. 2013). The importance of studying the combined effects of temperature and salinity to aquatic organisms has also been increasingly highlighted (Gagnaire et al. 2006; Munari et al. 2011). While there is substantial information available on the effect of single parameters on the metabolism and physiological processes of aquatic organisms, published reports on the combined effects of multiple parameters on aquatic animals, especially estuarine organisms, are still limited. Little is known about the combined effects of temperature and salinity on the metabolism of bivalve molluscs.

The relationship between body weight and metabolism is closely linked in bivalves (Vladimirova et al. 2003). Bivalves can dominate benthic biomass, coupling benthic and pelagic cycling processes (Dame and Patten 1981; Strayer et al. 1999; Vaughn and Hakenkamp 2008; Dame 2011). Bivalves are also considered to be optimal bioindicators for contamination by heavy metals and organic contaminants in aquatic environments (O’Connor 2002; Rigonato et al. 2005; Cheggour et al. 2005; Fedato et al. 2010). In recent years, because of water pollution, bivalve shellfish aquaculture has increased in some estuaries to monitor and reduce contamination from both water and sediment sources, e.g. clam and oyster cultivation in the west coast estuaries of North America (Dumbauld et al. 2009) and oyster production in the Yangtze River Estuary, China (Quan et al. 2007). *Corbicula fluminea* is of Asiatic origin, but has been introduced into many parts of the world, including North America and Europe (Sousa et al. 2008). This species is a prominent component of the benthic community in most Asian estuaries including the Yangtze River Estuary (Chen et al. 2005; Yang et al. 2006; An et al. 2007). *C. fluminea* has a high filtration capacity, excreting metabolic wastes and inorganic nutrients that can promote the growth of algae and enhance the energy flow of benthic communities. Aquaculture of these species and other bivalves can modify estuarine systems by biodeposition and bioturbation (Lauritsen and Mozley 1989; Phelps 1994; Hakenkamp et al. 2001; Karatayev et al. 2003; Sousa et al. 2008; Dumbauld et al. 2009; Menninger 2012). *C. fluminea* accumulates organic pollutants and heavy metals from both water and sediment sources (Doherty 1990). *C. fluminea* have been widely used as an effective biological indicator, mainly because of their widespread distribution, high fecundity, rapid growth rates and their 1-3 year life-span. These organisms are easy to collect because of their sedentary lifestyles and can readily adapt to experimental conditions in the laboratory (Doherty 1990; Menninger 2012). The basal metabolism (e.g. Oxygen consumption rate, ammonia excretion rate, and CO_2_ excretion rate) of the organism can be indexed to evaluate the extent of environmental disturbances (Tátrai 1982; Zheng et al. 2008). The influence of salinity and temperature on the metabolic processes of different size ranges of *C. fluminea* in the laboratory was used to establish species tolerance levels for these parameters. This information can be used to monitor variations within some estuaries, providing baseline data for restoration management programs.

## Materials and methods

### Animal collection and acclimation

*C. fluminea* were obtained by purchasing from a farm on Chongming Island, an alluvial island located on the Yangtze River Estuary, placed in a large container and transported to the laboratory within two hours. The clams were cleaned to remove any fouling and were acclimated in aerated 300 L plastic tanks, containing water at 21 ± 1°C with two salinities of 0.3‰ and 1.8‰, respectively.

### Experimental design

Prior to the start of the experiment, individuals of *C. fluminea* were measured and divided into three groups (small, medium and large) based on shell size (Table 1). Two salinity (0.3‰ and 1.8‰) were selected in the present experiment. Ten same size animals were transported into one 5 L glass container with filtered seawater at one salt level (0.3‰ or 1.8‰). Empty flasks filled with filtered seawater (without animals) were used as controls. Three replicates per treatment were measured. All flasks were sealed with liquid paraffin, to ensure that they were airtight. A preliminary experiment to determine the effects of body size and salinity on the metabolism of *C. fluminea* was conducted under controlled temperature conditions (21.9 ± 0.2°C). To observe the effect of any diurnal rhythm on the metabolic rate of the clams, oxygen consumption rate (OCR), ammonia excretion rate (AER), and CO_2_ excretion rate (CER) of *C. fluminea* were measured every 6 hours. After every experiment, a water sample from each flask was kept into an acid-washed polyethylene bottle and stored at -20°C.

**Table 1:** **Biological measurements of** ***Corbicula fluminea*** **used during the experiment**

Biological measurement	Large size/mm	Medium size/mm	Small size/mm
Shell length	28.194 ± 2.110	24.238 ± 1.514	19.310 ± 1.137
Shell height	25.514 ± 1.961	22.072 ± 1.333	17.701 ± 1.438
Shell width	17.084 ± 1.494	15.094 ± 1.129	12.884 ± 1.104
Soft tissue Dry weight	1.310 ± 0.219	0.903 ± 0.169	0.526 ± 0.071
Shell Dry weight	21.847 ± 2.627	15.132 ± 1.766	7.684 ± 0.665

Based on our previous experimental results only small size clams unaffected by salinity changes were used in the subsequent temperature experiment. We selected five temperature levels (4, 11, 18, 25 and 32°C) which cover the range of natural temperature variation throughout the year in most estuaries. Two salinity conditions (0.3‰ and 1.8‰) continued to be used during the experiment. Following a 24 hour acclimation period at each of ten salinity and temperature combinations, every ten similar sized clams were transferred into a respiration chamber (5 L transparent glass flasks) and then sealed with liquid paraffin. Control flasks without *C. fluminea* were treated similarly. Three replicates were performed per treatment. OCR, AER and CER of *C. fluminea* were measured at six-hour intervals for 24 hours.

Salinity and temperature were measured daily using a salinity pen (AZ-8371, Shenzhen Laesent Technology Co. Ltd.). To gain OCR, AER and CER of *C. fluminea* from every treatment group, all water samples were analyzed within 12 hours after being pushed into the acid-washed polyethylene bottle. Dissolved oxygen concentrations were measured in a 500 ml hermetic flask using a HACH-HQ30d oxygen meter with 0.01 mg/l accuracy. Ammonia-nitrogen concentrations were analyzed according to the Nessler’s reagent colorimetric method (Koch and McMeekin 1924; Vanselow 1940). CO_2_ concentrations were measured as described in Bundy and Bremner (1972). To minimize measurement errors, each treatment was analyzed 10 times. At the beginning and end of the experiment, the height, width, and length of shell were measured using a calibrated vernier micrometer. Each specimen was dissected and the shell and tissue dry weight measured to the nearest 1 mg.

OCR, AER and CER were calculated using the following equations (Cerezo Valverde et al. 2006):


The initial and final concentrations of dissolved oxygen (*DO*), ammonia-nitrogen (*N*) and CO_2_ (*C*) are denoted by subscripts *0* and *t*, respectively, *V* is the volume of respiration chamber (l), *DW* is the dry weight of *C. fluminea* and *T* is the time between the initial and final measurements (h).

The O:N atomic ratio (atoms of oxygen consumed per atom of N excreted) was used to estimate the proportion of protein in relation to lipids or carbohydrates for metabolism (Babarro et al. 2000):


The *Q*10 (temperature coefficient), a measure of the rate of change of a biological or chemical system as a consequence of increasing the temperature by 10°C, was calculated for *C. fluminea* according to the equation (Bayne and Newell 1983; Saucedo et al. 2004)


where *t*_*1*_ and *t*_*2*_ represented the temperature of two group trials respectively, *R*_*1*_ and *R*_*2*_ represented corresponding OCR under each temperature group.

### Data analyses

All data were expressed as mean ± standard deviation (SD) and were analyzed using SPSS17.0 (Windows statistical package). The assumption of homoscedasticity was determined before using parametric tests.The effects of different temperature and body size for each salinity were analyzed with one-way ANOVA. Differences between two salinities were analyzed with a *t*-test. Interactions between clam size and salinity and between salinity and temperature were analyzed with a two-way ANOVA. An alpha level of *p* < 0.05 was used to indicate significance of tests.

## Results

### Impact of salinity on metabolic rate

**Figure 1:**
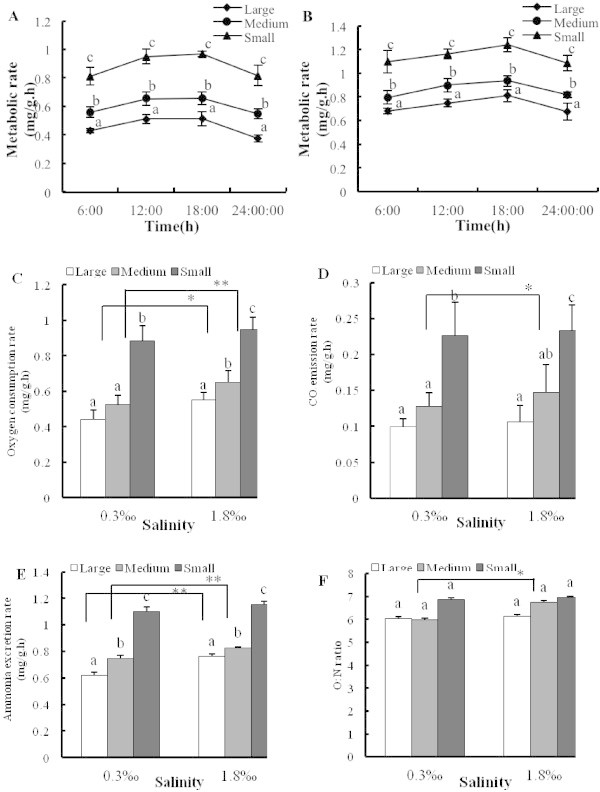
**Impact of salinity on three indexes of basal metabolism (OCR, AER and CER), patterns of diurnal rhythm and O:N ratios of three size** ***Corbicula fluminea.*** Diurnal rhythm of metabolic rate for three size ranges of *C. fluminea* at **(A)** 0.3‰ and **(B)** 1.8‰ salinity treatments, and effect of *C. fluminea* body size and salinity on **(C)** oxygen consumption rate, **(D)** CO_2_ emission rate, **(E)** ammonia excretion rate and **(F)** O: N ratio. Letters a, b and c are used to show significant differences between OCR, CER, AER and O: N ratio from the different sizes of *C. fluminea*, equal letters show not significantly different (one-way ANOVA).

The metabolic rates of the three size ranges of *C. fluminea* for two salinities are presented in Figure 1A and B. Similar diurnal trends were observed across both salinity regimes. The highest metabolic rate was observed during the evening (18:00), while the lowest metabolic rate was observed at midnight (24:00), each day. Small *C. fluminea* showed higher metabolic rates than medium and large *C. fluminea* (Independent-samples Test, *F* = 0.188, *P* = 0.046).

As shown in Figure 1C-E, there was a decrease in the OCR, CER and AER with increasing body size of *C. fluminea* at both salinities. Figure 1C showed that there was a significant difference between the two salinity values in the large size group of *C. fluminea* (Independent-samples Test, *F* = 0.239, *P* = 0.022) and a highly significant difference in the medium size (Independent-samples Test, *F* = 1.512, *P* = 0.006). For CER of *C. fluminea*, a significant difference was also found between two salinity treatments for medium size clams (Independent-samples Test, *F* = 0.197, *P* = 0.031) (Figure 1D). In Figure 1E, there was a highly significant difference in AER in both salinity groups for both large (Independent-samples Test, *F* = 5.895, *P* = 0.000) and medium-sized animals (Independent-samples Test, *F* = 20.708, *P* = 0.000). Overall, no statistically significant difference in OCR, CER, and AER was reported between salinity treatments for small *C. fluminea* (Independent-samples Test, *F*_OCR_ = 1.300, *P*_OCR_ = 0.311; *F*_CER_ = 1.102, *P*_CER_ = 0.892; *F*_AER_ = 1.591, *P*_AER_ = 0.603).

O: N ratios of the three size ranges at both salinity levels are shown in Figure 1F. The O:N ratio increased with increasing salinity for animals within the same size range, and there was no significant difference between two salinity treatments for the small and large sizes but a significant difference for the medium size range (Independent-samples Test, *F* = 1.671, *P* = 0.734). Under the same salinity conditions, no significant difference of O: N ratios was found for three different-sized *C. fluminea* (Figure 1F).

### The impact of temperature on metabolic rate

**Figure 2:**
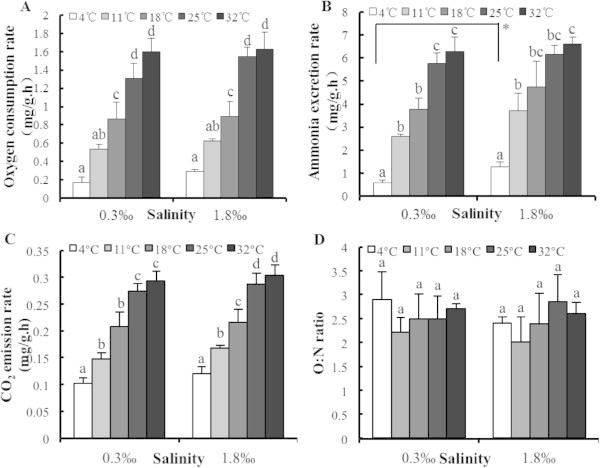
**Effect of temperature and salinity on (A) oxygen consumption rate, (B) ammonia excretion, (C) CO**_**2**_ **emission rate and (D) O:N ratio for small** ***C. fluminea.*** Letters a, b and c are used to show significant differences between OCR, CER, AER and O: N ratio from the different temperatures of *C. fluminea*, equal letters show not significantly different (one-way ANOVA).

For the group of small clams, the variation of all three basal metabolism indices and temperatures are shown in Figure 2A-C. These three indicators increased significantly with increasing temperature from 4°C to 25°C, but no significant difference was found between the 25 and 32°C treatments. Between two salinity group, there was no significant difference for three indicators except for the AER of *C. fluminea* at 4°C (Independent-samples Test, *F* = 1.729, *P* = 0.004) (Figure 2B).

O: N ratios at the different temperature and salinity conditions for small *C. fluminea* are shown in Figure 2D. At the lower salinity (0.3‰), the highest and lowest O: N ratio occurred at 4°C and 11°C, respectively. At the higher salinity (1.8‰), the highest O: N ratio was at 25°C, with the lowest O: N ratio occurring at 11°C. However, there was no significant difference in the O: N ratios among the five temperatures and two salinity treatments.

For the small-sized *C. fluminea*, the *Q10* coefficients for different temperatures and salinities are shown in Table 2. The *Q10* coefficient for the 1.8‰ salinity treatment was lower than for the 0.3‰ salinity treatment at the same temperature ranges, but no significant difference existed. In both salinity groups, the highest *Q*_*10*_ coefficients (*Q*_*10*_ = 1.825 at salinity of 0.3‰ and *Q*_*10*_ = 1.683 at salinity of 1.8‰) were observed at the 18-25°C temperature treatments. The low values of *Q*_*10*_ were found in the 4-11°C, 11-18°C and 25-32°C temperature treatments for the 0.3‰ and 1.8‰ salinity treatments, respectively.

**Table 2:** **Mean values (±S.D) of the Q**_**10**_ **coefficient in** ***C. fluminea*** **at different temperature**

Salinity	Temperature	N	Q _10_coefficient
0.3‰	4-11°C	10	0.732 ± 0.094
	11-18°C	10	0.786 ± 0.291
	18-25°C	10	1.825 ± 0.412
	25-32°C	10	0.741 ± 0.489
1.8‰	4-11°C	10	0.650 ± 0.099
	11-18°C	10	0.695 ± 0.296
	18-25°C	10	1.683 ± 0.234
	25-32°C	10	0.170 ± 0.119

N = number of animals.

### Influence of body size, salinity, and temperature on metabolic rate

The interaction between clam size and salinity had a highly significant difference for AER (two-way ANOVA, *F* = 50.347, *P* = 0.001), but no significant differences were noted for the other metabolic indices (Table 3). There were no significant differences based on the interaction between salinity and temperature and the basal metabolic measurements (two-way ANOVA, *F*_OCR_ = 0.222, *P*_OCR_ = 0.923; *F*_AER_ = 0.164, *P*_AER_ = 0.954; *F*_CER_ = 0.236, *P*_CER_ = 0.915; *F*_O:N_ = 0.182, *P*_O:N_ = 0.945).

**Table 3:** **The interactive influence of** ***C. fluminea*** **size and the exposure salinity and temperature on OCR, AER, CER and the O:N ratio**

Metabolic index	Size Salinity				Salinity Temperature			
	***df***	***MS***	F value	P value	***df***	***MS***	F value	P value
Oxygen consumption rate	2	0.002	0.598	0.561	4	0.011	0.222	0.923
Ammonia excretion rate	2	0.004	50.347	0.001	4	0.177	0.164	0.954
CO_2_ emission rate	2	0.000	0.045	0.956	4	0.000	0.236	0.915
O:N ratio	2	0.438	0.788	0.470	4	0.140	0.182	0.945

*df* = degree of freedom, *MS* = Mean Square.

## Discussion

### Effect of body size on the metabolism of *C. fluminea*

The body size of bivalve molluscs is an important parameter strongly correlated with respiration, excretion and clearance rates (Yukihira et al. 1998; Matthews and McMahon 1999; Taware et al. 2012). For all three-size ranges of *C. fluminea*, a similar diurnal rhythm was observed with the highest and lowest rates observed respectively at 18:00 and 24:00 regardless of size. This characteristic has been reported in other bivalve species, such as *Katelysia opima* and *Soletellina diphos* (Mane 1975; Taware et al. 2012). Our results also showed that OCR, AER and CER decreased with increasing body size in *C fluminea*. In particular, the metabolic rate of the small group was distinctly higher than for the large and medium-sized animals. This suggested that smaller clams were more metabolically active than the large ones. This result is consistent with other bivalve species, e.g. *Dreissena polymorpha* and *D. bugensis* (Summers et al. 1996), and *Soletellina diphos* (Taware et al. 2012). Majdi et al. (2014) also observed that small *C. fluminea* showed the highest net sediment-reworking rate. Mane (1975) stated that body size was an important factor to change the metabolic rate in bivalves; hence, the older and large individuals had a lower metabolic rate than small individuals. Jadhav et al. (2012) showed that the energy flow through smaller individuals of a species could be much greater than that of larger individuals. O: N ratio is an effective index for assessing the contribution of protein catabolism to total metabolism and can provide indices of balance between the catabolism rates of proteins, carbohydrates, and lipid substrates in animal tissues (Jadhav et al. 2012). High O: N ratio points out catabolism of carbohydrate and lipid (Bayne 1976), while low O: N ratio rather points out protein catabolism (Mace and Ansell 1982). The variation in values of the O: N ratio with body size was typical for oxygen consumption and ammonia excretion rates (Stickle and Bayne 1982). Larger organisms show higher protein catabolism (Gabbott and Bayne 1973), this trend is supported by our results: we showed that smaller individuals presented higher values of O: N ratio than larger ones, which is consistent with results for other bivalves, for example, *Lamellidens marginalis* (Jadhav et al. 2012) and *Soletellina diphos* (Lagade et al. 2013). The results suggest that the production of small clams is oriented towards gaining tissue biomass (carbohydrates and lipids), whereas larger clams oriented their production towards proteic catabolism (e.g. gametogenesis).

### Effects of salinity on the metabolism of *C. fluminea*

In this study, the values of O:N ratio ranged from 6.025 at 4°C (0.3‰) to 8.686 at 11°C (1.8‰), these low O: N ratios indicate that protein was a primary metabolic substrate because of a relatively small glycogen reserve (Babarro et al. 2000; Lagade et al. 2013). For the O: N ratios of small *C. fluminea*, no significant differences for small clams were found across five water temperatures and two salinities, which shows small clams could well adapt to temperature changes of the Yangtze River Estuary (4 ~ 30°C).

Sousa et al. (2008) concluded that *C. fluminea* could tolerate salinities ranging from 0‰ to 5‰. In this study, two salinity levels within this range were selected based on the salinity profile of the Yangtze River Estuary (0.3 ~ 1.8‰) (Mao et al. 2001). The results showed that the metabolic rate of our same-sized clams was higher at a salinity of 1.8‰ than 0.3‰; despite a significant difference between both salinity types for the medium and large size groups, there were no significant difference for the small size group (Figure 1C-E). It’s similar to our O: N results in this study (Figure 1F). This suggests that *C. fluminea* are increasingly affected by salinity as they grow and large clams have lower tolerance than small ones to fluctuations in the Yangtze River Estuary. In contrast, Soria et al (2007) show that juvenile scallops *Argopecten purpuratus* were more sensitive to low salinity and demonstrated lower survival than larger animals. In other words, this discrepancy makes sense since *A. purpuratus* is adapted to marine conditions while *C fluminea* is a largely freshwater species (Soria et al. 2007).

Christophersen and Strand (2003) reported a clear synergetic effect of temperature and salinity on growth of *Pecten maximus*. In our study, this synergetic effect of the two factors was not found on the metabolic rate of small *C. fluminea* (Table 3). However, a strong positive correlation was found between temperature and the metabolic rate from 4°C to 25°C for the small-sized *C. fluminea*, but no significant differences at the same temperature treatment were observed between the two salinities (Figure 2A-C). This implies that the small *C. fluminea* had a higher tolerance to the low salinity changes when compared to medium and large size groups. This ability could allow them to become a dominant species under similar environmental conditions in most estuaries of Southeast Asia (Chen et al. 2005; Yang et al. 2006; An et al. 2007). In ecosystem restoration of an estuary, the survival rate of *C. fluminea* would be improved by releasing the small size one, which can contribute to effectively conserve or restore ecological function by biodeposition and bioturbation.

### Effect of temperature on the metabolic rate of small-sized *C. fluminea*

Metabolic rate in bivalves can vary considerably with size and the complex interactions of seasons and temperature (Bayne and Scullard 1977). In this study, the experimental temperatures between 4°C and 32°C covered the range of natural temperature variation for the Yangtze River Estuary (4 ~ 30°C) and were selected to find out whether *C. fluminea* could maintain a normal metabolic rate here. Some previous reports suggested that rates of metabolism increase directly with increasing temperature and then rapidly decrease when an optimal limit is reached. This pattern has been supported in most species of bivalve molluscs (Paul 1980; Saucedo et al. 2004). In this study, three proxies of *C. fluminea* metabolic activity (OCR, AER and CER) showed significant increase with temperature rising from 4°C to 25°C. Metabolic rate optimum was not found, though a plateau was observed between 25-32°C. The metabolic rate of small *C. fluminea* remained high at the plateau stage. It is apparent that the temperature have not reached maximum enough to make their metabolic damage, to small *C. fluminea*.

The *Q*_*10*_ coefficient has been identified as an index relating to enzymatic and physiological requirements for energy, when a 10°C temperature increases within an organism’s tolerance range (Zheng et al. 2008). In this study, *Q*_*10*_ values for OCR were calculated at the 7°C intervals for small *C. fluminea*. No significant difference between the two salinities were found in the same temperature interval. However, low temperature coefficients were found at three different temperature intervals (between 4 and 11°C, between 11 and 18°C and between 25 and 32°C), and an increase of more than double at the middle 18-25°C interval. This suggest that small *C. fluminea* experience little energy loss at the lower (4-11°C and 11-18°C) and upper (25-32°C) temperature ranges and is capable of performing seasonal compensation to maintain their capacity to survive in their range of temperature tolerance. An adequate metabolic temperature range for small *C. fluminea* may lie between 18 and 25°C because of significantly increasing OCR with the increasingly temperature. Similar results were reported in *Pinctada mazatlanica* (Saucedo et al. 2004). So Saucedo et al. (2004) suggested that some bivalves not only acclimate well to temperature changes so that physiological processes of clearance rates and respiration rates remain relatively independent of temperature, but also maintain high levels of energy balance over relatively broad temperature ranges.

## Conclusion

*C. fluminea* has been increasing cultivated in some Asian estuaries (e.g. the Yangtze River Estuary) because of their ability to monitor and reduce contamination (e.g. organic pollutants and heavy metals) from both water and sediment sources. Variations of temperature and salinity produced by ocean tides and freshwater rivers are a major characteristic in estuaries, which affect survival rate of *C. fluminea*. Based on our findings, it is concluded that: (1) a narrow range of salinity (0.3 and 1.8‰) has little effect on the metabolism of small-size *C. fluminea*; (2) a temperature of 18-25°C may represent an optimum adequate metabolic temperature range for small clams and they may compensate for temperature changes at the lower (4-11°C and 11-18°C) and upper (25-32°C) ranges. Our results indicate that *C. fluminea* thus survive well in many estuaries (including the Yangtze River Estuary), and that small clams have a greater ability to adapt to variations of salinity and temperature. When clams are planted for the purposes of ecological monitoring and restoration, small individuals of *C. fluminea* are more likely to survive than larger ones.
